# Supplementary material for: Rheological, Thermal and Mechanical Characterization of Toughened Self-Healing Supramolecular Resins, Based on Hydrogen Bonding
Source: Nanomaterials (Basel). 2022 Dec 5;12(23):4322. doi: 10.3390/nano12234322 (PMC9735688; doi:10.3390/nano12234322)
Supplement: Supplementary file 1 [file nanomaterials-12-04322-s001.zip › nanomaterials-2004807-supplementary.pdf]

nanomaterials

MDPI

*Supplementary Materials*

# Rheological, Thermal and Mechanical Characterization of Toughened Self-Healing Supramolecular Resins, Based on Hydrogen Bonding

**Liberata Guadagno [1,*], Marialuigia Raimondo [1], Carlo Naddeo [1], Luigi Vertuccio [2], Salvatore Russo [3], Generoso Iannuzzo [3] and Elisa Calabrese [1]**

[1] Department of Industrial Engineering, University of Salerno, Via Giovanni Paolo II, 84084 Fisciano, Italy
[2] Department of Engineering, University of Campania "Luigi Vanvitelli", Via Roma 29, 81031 Aversa, Italy
[3] Leonardo Aircraft Division, Viale Dell'aeronautica, 80038 Pomigliano d'Arco, Italy
* Correspondence: lguadagno@unisa.it

## Rheological measurements

Rheological measurements of the functional uncured formulations were performed with a Physica-MCR301 (Anton-Paar) rotational rheometer. Small amplitude oscillatory shear measurements were performed at different values of temperatures.

## Electrical properties

DC volume conductivity measurements were carried out by using disk-shaped samples of about 2 mm thickness and 50 mm diameter; using a circular electrode with a diameter of about 22 mm. The system employed for the measurements was remotely controlled by the software LABVIEW®. It is constituted of a shielded cell with the control of temperature, of multimeter Keithley 6517A with the function of voltage generator (max. _1000 V) and voltmeter (max. _200 V) and the ammeter HP34401A (min. current 0.1 mA) for specimens above the EPT. For samples below the EPT, the system is constituted only of multimeter Keithley 6517A with the function of voltage generator (max. _1000 V) and pico-ammeter (min. current 0.1 fA).

## Self-healing efficiency evaluation

Figure S1 illustrates the samples with TDCB geometry clamped and located in the dynamometer. Samples have been loaded at first failure and then unloaded, allowing the crack face to come back into contact and after 24 h have been tested again.

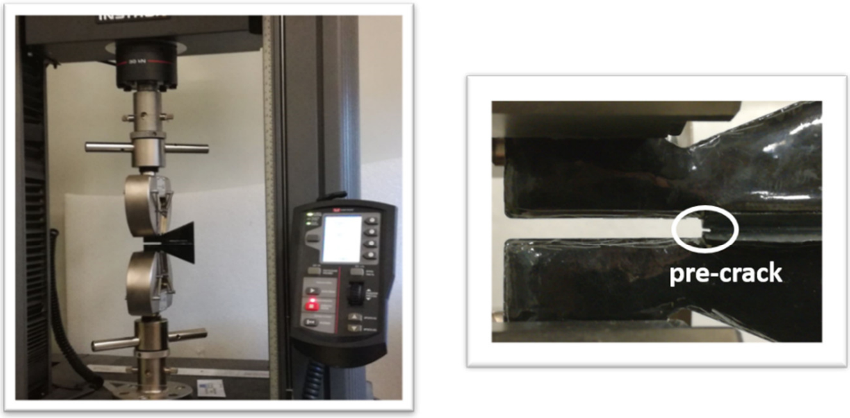


**b)** **c)**

**Figure S1.** (**a**) Virgin sample (EP-*0.5*CNT-M) with TDCB geometry located in the instrument for fracture tests; (**b**) enlargement of photo a, showing the pre-crack performed on the sample before the test.

## Rheological characterization

**Table S1.** Values of complex viscosity (η*) at different temperatures for EP blend.

| Temperature [°C] | η* [Pa*s] |
|---|---|
| 25 | 1726.552 |
| 50 | 22.89545 |
| 75 | 1.668182 |
| 90 | 0.610136 |

**Table S2.** Values of complex viscosity (η*) at different temperatures EP blend loaded with healing molecules.

| T [°C] | η* EP-DBA [Pa*s] | η* EP-T [Pa*s] | η* EP-M [Pa*s] |
|---|---|---|---|
| 25 | 11100 | - | 2550 |
| 50 | 92,8 | 24,5 | 37,6 |
| 75 | 6,43 | 2,04 | 2,63 |
| 90 | 2,87 | 0,77 | 1,02 |

## Thermal analysis

**Table S3.** TGA data of cured samples in air and nitrogen flow.

| | TGA in Air Flow | | | TGA in Nitrogen Flow | | |
|---|---|---|---|---|---|---|
| Sample | $Td_{5\%}$ | $Td_{50\%}$ | Residue (900 °C) | $Td_{5\%}$ | $Td_{50\%}$ | Residue (900 °C) |
| EP | 363.3 | 444.4 | 2.6 | 348.4 | 411.1 | 18.2 |
| EP-*0.5*CNT | 356.0 | 444.3 | 0 | 338.6 | 413.3 | 20.0 |
| EP-*0.5*CNT-DBA | 365.8 | 453.3 | 4.2 | 359.2 | 413.0 | 20.0 |
| EP-*0.5*CNT-T | 316.1 | 435.2 | 0 | 340.8 | 413.6 | 9.8 |
| EP-*0.5*CNT-M | 335.4 | 454.5 | 2.7 | 336.2 | 431.7 | 9.8 |
